# Morning blood pressure surge in early autosomal dominant polycystic kidney disease and its relation with left ventricular hypertrophy

**DOI:** 10.1080/0886022X.2020.1864403

**Published:** 2021-01-21

**Authors:** Abdülmecit Yildiz, Saim Sag, Cuma Bulent Gul, Sümeyye Güllülü, Fatma Ezgi Can, Ömer Bedir, Mehmet Fethullah Aydin, Ayşegül Oruç, Sadettin Demirel, Suat Akgür, Mustafa Güllülü, Alparslan Ersoy

**Affiliations:** a Department of Nephrology, Bursa Uludag University Faculty of Medicine, Bursa, Turkey; b Department of Cardiology, Bursa Uludag University Faculty of Medicine, Bursa, Turkey; c Department of Nephrology, Bursa Yuksek Ihtisas Training and Research Hospital, Bursa, Turkey; d Department of Biostatistics, Bursa Uludag University Faculty of Medicine, Bursa, Turkey; e Department of Physiology, Bursa Uludag University Faculty of Medicine, Bursa, Turkey

**Keywords:** Autosomal dominant polycystic kidney disease, left ventricular hypertrophy, endothelial dysfunction, morning blood pressure surge

## Abstract

**Introduction:**

The activation of the sympathetic nervous system, which usually leads to a swift surge in blood pressure in the morning hours (MBPS) may be the cause of left ventricular hypertrophy (LVH) and endothelial dysfunction (ED) in early autosomal dominant polycystic kidney disease (ADPKD) patients. We studied the association between MBPS and LVH in ADPKD patients with preserved renal functions.

**Methods:**

Patients with ADPKD with preserved renal functions were enrolled. Prewaking MBPS was calculated using ambulatory blood pressure monitoring. The patients were categorized as MBPS (≥median) and non-MBPS (<median). Left ventricular mass index (LVMI), endothelial-dependent dilatation (FMD, %), and carotid intima-media thickness (CIMT) evaluated.

**Results:**

Fifty-six patients (30 females and 26 males) were enrolled. Gender distribution was similar-among-the-groups. The mean age was higher in the MBPS group (50.1 ± 13 vs 37.3 ± 10.3). Urinary albumin (49.5 vs 16 mg/g creatinine, *p* < 0.001), hs-CRP (0.59 vs 0.37 mg/dl, *p* = 0.045) LVMI (124 ± 27.7 vs 95.2 ± 19.7 g/m^2^, *p* < 0.001) and mean awake SBP surge was higher (42 vs 20 mmHg, *p* < 0.001) and FMD (%) was lower (14.4 ± 6.6 vs 18.9 ± 5.7, *p* = 0.009) in MBPS group. In the binary logistic regression analysis, the presence of MBPS in model 1 (OR: 6.625, 95% CI [1.048–41.882] *p* = 0.044), and age in model 2 (OR: 1.160, 95% CI [1.065–1.263] *p* = 0.001) were the only independent determinant of LVH.

**Conclusions:**

MBPS seems to be an important and independent determinant of LVH in ADPKD patients with preserved renal functions. It may be worth assessing the effect of reduction in MBPS as a new therapeutic target to prevent LVH in-patients-with-ADPKD.

## Introduction

Autosomal dominant polycystic kidney disease (ADPKD) is the most common hereditary kidney disease and is characterized by extra-renal manifestations ranging from liver disease to various cardiac abnormalities such as mitral valve prolapses [1]. In addition, some cardiovascular (CV) abnormalities like left ventricular hypertrophy (LVH) and endothelial dysfunction (ED) have been reported in young normotensive ADPKD patients with preserved kidney function. However, in normal populations, both LVH and ED are commonly found in hypertensive and elderly patients but not in normotensive and young individuals [2]. Although some pathophysiologic events such as borderline hypertension (HT), increased sympathetic activity, and abnormal ciliary activity on endothelial cells have been suggested as a cause of LVH and ED in early ADPKD patients, no clear cause has been identified until now [3]. Some studies on ADPKD patients evaluated the 24-h ambulatory blood pressure (BP) characteristics and found impaired circadian variation in BP [4,5]. However, to our knowledge, no study sought MBPS and Its relation with CV abnormalities, which are common in ADPKD patients. Morning hours are characterized by the highest incidence of major cardiovascular events, including myocardial infarction, stroke, or sudden death. The most likely reason for this is the activation of the sympathetic nervous system in the early hours of the day leads to a rapid increase in blood pressure (BP), known as MBBS. Chronically higher levels of MBPS may result in structural alterations in arterial vessels lead to LVH in the myocardium and ED in the vascular bed.

Interestingly, even in the normotensive population, the isolated elevation of morning BP has been found associated with left ventricular hypertrophy [6]. This impaired variation may partly explain CV abnormalities that are seen in ADPKD. Interestingly, in light of these observations, some studies found a relation between MBPS and CV morbidity [6] and LVH [7]. However, although ED and LVH are common in the absence of prominent HT in early ADPKD patients, there has been no investigation of the association between elevated MBPS and these abnormalities in ADPKD patients. We aimed to investigate the association between waking MBPS and LVH in ADPKD patients with preserved renal functions. We also aimed to assess the association of MBPS with ED in these patients.

## Subjects and methods

### Study design

This cross-sectional study took place in the nephrology clinic of Bursa Uludag University Faculty of Medicine.

### Study population

A total of 114 ADPKD patients from the Caucasian population who were diagnosed based on positive family history for ADPKD and renal ultrasonography findings [8] were consecutively evaluated at the nephrology outpatient clinic of Bursa Uludag University School of Medicine between July 2013 and February 2016. Patients suffering from established CV diseases including congestive heart failure, coronary artery disease, stroke, diabetes mellitus (DM), pregnant women, patients who had uncontrolled hypertension or were hypertensive during an outpatient visit (SBP ≥140 mmHg and/or DBP ≥90 mmHg) were excluded. Hypertension with controlled at least two drugs, diabetes, and established CV diseases was excluded because they are closely related to LVH, which was the primary study outcome. Patients were using losartan or ramipril as antihypertensive drugs (6/6 in the MBPS group and 7/5 in the Non-MBPS group. We aimed to identify patients who are clinically not having apparent CV disease but have diminished circadian rhythm of BP that could adversely affect the heart (LVH) or the vascular system (ED).

### Study variables

The primary exposure was MBPS, and the primary outcome was LVH and ED. Secondary outcomes were other CV characteristics, including carotid intima-media thickness (CIMT) and left ventricular geometry. After providing detailed medical history and undergoing a physical examination, each participant was questioned for major cardiovascular risk factors such as age, sex, DM, smoking status, HT, and medications. Venous blood samples for biochemical analyses were taken after an overnight fast. Glucose, creatinine, uric acid, and lipid profiles were determined using standard methods. High-sensitivity CRP (hs-CRP) was measured using the nephelometric method (Siemens BNII, Cardiophase, Germany). The estimated glomerular filtration rate (eGFR) was computed by CKD-EPI (Chronic Kidney Disease Epidemiology Collaboration) and the creatinine equation, as defined by Levey et al. [9]. Spot urine albumin and creatinine measurements were performed, and urinary albumin/creatinine ratios (ACr) were calculated. Microalbuminuria was defined as an ACr value > 30 in the spot urine.

### Ambulatory blood pressure measurements and the calculation of morning blood pressure surge

The 24-h BP monitoring was performed using a Contec-branded device (ABPM50, Germany), and the results were analyzed using the manufacturer’s computer software. Ambulatory measurements were carried out once every 30 min from 7 am until 11 pm and once every 60 min from 11 pm until 7 am. An evaluation was performed by taking the mean values of day and night blood pressures into account. HT was considered to be present if the average systolic blood pressure (SBP) was 135 mmHg and/or the average diastolic blood pressure was 85 mmHg during the entire day. MBPS can be calculated by two way with different thresholds such as sleep-trough (>55 mm Hg, presence of MBPS) and prewaking MBPS (>34 ± 21 mm Hg presence of MBPS) defined by Kario et al. [10]. We used in our study prewaking MBPS and calculated as the average SBP during the first 2 h after wake-up time (4 BP readings), pre-awake SBP was defined as the average SBP during the 2 h just before wake-up time (4 BP readings), and the difference was calculated (prewaking MBPS). A difference greater than the median value of the study group (26 mmHg) was accepted presence of MBPS (MBPS group). A universal cutoff for defining elevated MBPS is missing and in European subjects, MBPS is on average lower [11]. Some studies divided their patients with respect to their MBPS medians or quartiles [12,13]. So, we used the median MBPS to compare subjects with and without MBPS in terms of the study outcomes. Subjects whose nocturnal decline in SBP was ≥10% of their daytime SBP were classified as dippers, and subjects whose nocturnal decrease in SBP was <10% of their daytime SBP were classified as nondippers. The SBP was used for all these calculations.

### Echocardiography investigation

Standard 2 D and Doppler transthoracic echocardiography were performed with a widely available transducer and equipment (M3S probe, Vivid 7, GE-Vingmed, Horten, Norway) by standard techniques with the subjects at rest in the left lateral decubitus position. All echocardiographic measurements were performed based on the American Society of Echocardiography Guidelines (ASE) [14]. During the echocardiography, a one-lead electrocardiogram was recorded continuously. Left ventricular mass (LVM) was calculated according to the ASE-recommended formula: LVM = 0.8*x* + 0.6. Thereafter, the LVM index was obtained using the following formula: LVM/body surface area. Relative wall thickness (RWT) was measured at end diastole as the ratio of (2xLV posterior wall thickness)/left ventricular diameter (LVDd). LVH was defined as LVMI values >125 g/m2 in men and >110 g/m2 in women. Increased RWT was accepted as ≥0.45.

### The pattern of left ventricular geometry

Geometric patterns were based on the upper normal limits for LVMI and RWT; (I) normal geometry (NLV) (normal LVMI and normal RWT), (II) concentric remodeling (CR) (normal LVMI and increased RWT), (III) eccentric hypertrophy (EH) (increased LVMI and normal RWT), and (IV) concentric hypertrophy (CH) (increased LVMI and increased RWT).

### CIMT and ED

CIMT was measured from 10 mm proximal to the right common carotid artery bifurcation segment using the same device with a 12 MHz linear-array imaging probe. CIMT was calculated as the distance between the lumen-intima and media–adventitia interfaces. Flow mediated dilatation (FMD) of the brachial artery as surrogate marker of ED was measured according to the American College of Cardiology Guidelines. Briefly, subjects were allowed to rest for 20 min in a supine position in a semi darkened room at 23 °C. The left arm was positioned on a table at heart level. The resting diameter of the right brachial artery was measured 3–5 cm above the antecubital fossa using the same device with a 12 MHz linear-array imaging probe. Then, a blood pressure cuff was inflated around the right forearm to at least 50 mmHg above the systemic blood pressure for 4–5 min. Sixty seconds after cuff release, the diameter of the brachial artery was measured. The diameter change caused by FMD was expressed as the percentage change relative to that at the initial resting scan (FMD %).

### Statistical analysis

SPSS Statistics for Windows, Version 21 (IBM Corp., Armonk, NY) was used for statistical analysis. Descriptive statistics were reported as the mean ± standard deviation for variables with normal distribution and median (min–max) for variables with skewed distribution. Categorical variables were presented with frequency and percentage. Student’s *t*-test, Mann–Whitney *U* test, Pearson chi-square, and Fisher’s tests were used for intergroup comparisons. The relationship between continuous variables was determined by Pearson or Spearman correlation coefficients as needed. A value of *p* < 0.05 was considered statistically significant.

### Regression analysis

Clinically relevant factors that could affect LVH were included in binary logistic regression analysis. These factors were age, GFR, average SBP, MBPS, and dipper status. Because of the multicollinearity between age and GFR (*r* = 0.79), eGFR was excluded from the regression model. There was also a high correlation between MBPS and dipper status (*r* = 0.6); thus, two separate models, one including MBPS and the other, including dipper status, were used. Age and average SBP were used in both models.

## Results

**Figure 1.**
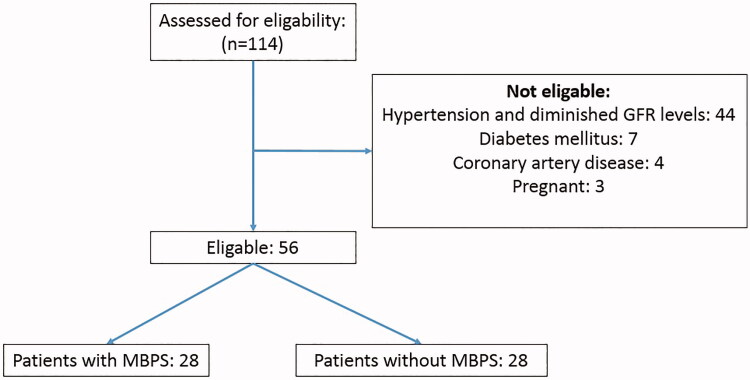
Flow diagram showing the quantitative details of patient enrollment.

A total of 114 ADPKD patients were evaluated, and 44 of them were excluded because they had diminished renal functions (CKD-EPI eGFR), uncontrolled hypertension, or required at least two antihypertensive drugs for BP control. Seven patients with diabetes, four patients with coronary artery disease, and three patients who were pregnant were also excluded from the study (Figure 1). The remaining 56 patients (30 female, 26 male) were enrolled in this study.

### Association between MBPS and study variables

Patients with MBPS were older and had higher hs-CRP, ACr, RWT, and LVMI values (Table 1). Among BP measurements, only awake mean SBP was higher in the MBPS group, and other averages, awake, and asleep BP measurements were similar. The MBPS group had a higher rate of LVH and was more commonly dippers (Table 1). FMD was significantly lower in the MBPS groups compared with the non-MBPS group. CIMT value tended to be higher in the MBPS group, but the difference was not statistically significant. The remaining study variables were similar between patients with and without MBPS.

**Table 1.** Clinical, laboratory, echocardiography and 24-h Holter BP features of study groups.

	MBPS group (*n* = 28)	Non-MBPS group (*n* = 28)	*p* Value
**Age**^a^	**49.5 ± 12.7**	**37.1 ± 10.4**	**<0.001**
Gender (%males)	39.3	53.6	0.4
Smoking (%)	39.3	32.1	0.8
RAS blocker use (%)	42.9	42.9	1
**Dipper (%)**	**67.9**	**32.1**	**0.008**
BMI (kg/m^2^)^a^	26.4 ± 4.7	25.8 ± 4.3	0.6
Fasting blood glucose (mg/dl)^b^	88 (74-106)	86.5 (72-129)	0.9
GFR-EPI (ml/min)^a^	96.1 ± 17.7	102.9 ± 22.4	0.2
**hsCRP (mg/dl)**	**0.59 (0.3-1.2)**	**0.37 (0.2-4)**	**0.045**
Albumin (g/dl)^a^	4.2 ± 0.4	4.3 ± 0.3	0.09
Uric acid (mg/dl)^b^	5.1 (3.2-9.5)	5.4 (2.2-9.9)	0.5
HDL-cholesterol (mg/dl)^a^	46.7 ± 9.5	42.1 ± 9.4	0.08
LDL-cholesterol (mg/dl)^a^	123.1 ± 32.3	117.4 ± 32.5	0.5
Triglyceride (mg/dl)^b^	132 (44-376)	117 (44-536)	0.6
Hemoglobin (g/dl)^a^	13.8 ± 1.4	14 ± 1.8	0.6
Albuminuria* **(** mg/g creatinine**)**	**49.5 (4-574)**	**16 (4-151)**	**<0.001**
**FMD (%)**^a^	**14.4 ± 6.6**	**18.9 ± 5.7**	**0.009**
**LVMI (g/m^2^)**^a^	**124 ± 27.7**	**95.2 ± 19.7**	**<0.001**
**LVH (%)**	**57.1%**	**7.1%**	**<0.001**
CIMT (mm)^b^	7 (3–13)	6 (3–12)	0.08
Mean SBP (mmHg, 24 hours)^a^	127.4 ± 14.7	121.1 ± 13.2	0.1
Mean DBP (mmHg, 24 hours)	77 (51-90)	74 (56-99)	0.3
Mean pulse (per minute, 24 hours)^a^	72 ± 9.7	75.3 ± 8.4	0.17
**Mean SBP (mmHg, awake)**^a^	**131.5 ± 16.8**	**123 ± 13.8**	**0.035**
Mean DBP (mmHg, awake)^b^	80 (52-94)	75.5 (58-94)	0.11
Mean SBP(mmHg, asleep)^a^	115.1 ± 15.2	115.2 ± 13.2	0.97
Mean DBP (mmHg, asleep)^a^	67.9 ± 9.7	68.9 ± 11.8	0.72
**MBPS (mmHg)**^b^	**42 (27-65)**	**20 (11–25)**	**<0.001**

BP: blood pressure; MBPS: morning blood pressure surge; BMI: body mass index; hsCRP: high sensitive C-reactive protein; HDL: high-density lipoprotein; LDL: low-density lipoprotein; FMD: flow-mediated dilatation; LVMI: left ventricular mass index; CIMT: carotid intimal media thickness; SBP: systolic blood pressure; DBP: diastolic blood pressure; RWT: relative wall thickness.

*Spot urine.

^a^Mean ± standard error of mean.

^b^Median (IQR).The bold values represents as statistically significant *p* <.05 values.

### Association between LVH and study variables

Patients with LVH tended to be more commonly female (66.7% vs. 47.4%, *p* = 0.18), smokers (50% vs. 28.9%, *p* = 0.13), and RAS blocker users (61.1% vs. 34.2%, *p* = 0.06), but these differences were not statistically significant. Patients with or without LVH had a similar frequency of dipper status (61.1% vs. 44.7%, respectively, *p* = 0.3). Mean age (54.9 ± 8.5 vs. 37.8 ± 11.2, *p* = 0.009), average SBP (131.3 ± 14.8 vs. 120.8 ± 12.8, *p* = 0.009), BMI (28.4 ± 3.8 vs. 25.1 ± 4.3, *p* = 0.008), HDL-cholesterol (48.3 ± 9.3 vs. 42.6 ± 9.4, *p* = 0.04) were higher, and GFR (91.6 ± 16.7 vs. 103.3 ± 20.9, *p* = 0.04) was lower in patients with LVH compared with those without LVH. ACr (85.6 ± 59.9 vs. 41.2 ± 93.3, *p* = 0.07) and LDL-cholesterol (129.1 ± 31.7 vs. 116 ± 32, *p* = 0.16) tended to be higher in patients with LVH, but the difference was not statistically significant.

### Distribution of left ventricular geometric patterns

**Figure 2.**
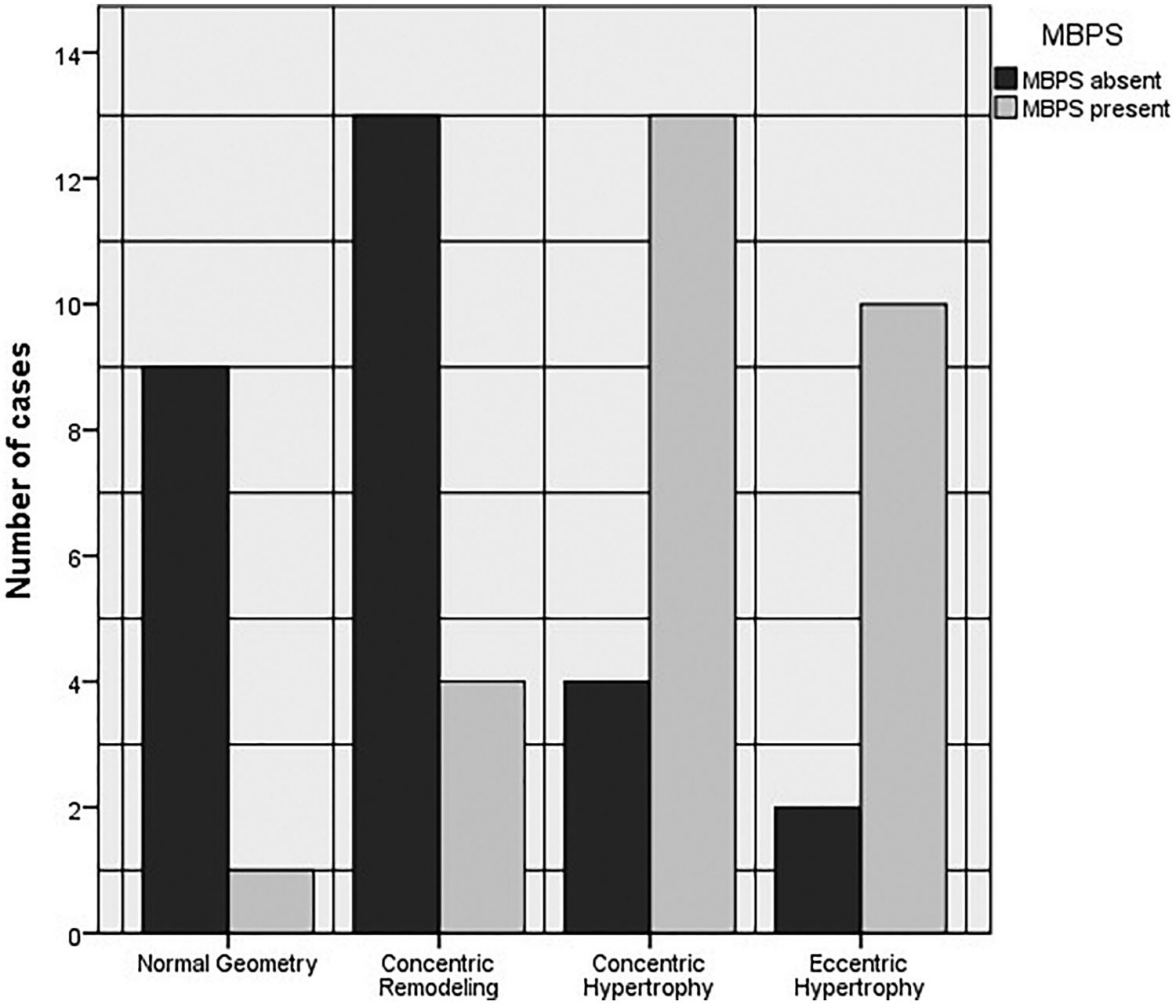
The distribution of left ventricular geometric pattern in MBPS and non-MBPS groups.

The patients in the MBPS group had a significantly higher frequency of concentric and eccentric LVH compared with those in the non-MBPS group (Figure 2). The frequency of the eccentric LVH and concentric LVH was 35.7% vs. 7.1% and 46.4% vs. 14.3% in the MBPS and non-MBPS groups, respectively (*p* < 0,001). Normal geometry and concentric remodeling were seen in 3.6% vs. 32.1% and 14.3% vs. 46.4% of the patients in MBPS and non-MBPS groups, respectively.

### Multivariate regression analysis

In the first model, the presence of MBPS (OR 6.625, 95%CI 1.048–41.882, *p* = 0.044) was independent predictors of LVH. THE average SBP was not independently associated with LVH (Table 2). The r^2^ of the model was 0.623, and Homer Lemeshow goodness-of-fit test indicated the model was a good fit (*p* = 0.17). In the second model, only age (OR 1.160, 95%CI 1.065–1.263, *p* = 0.001) was independently associated with LVH. The average SBP and dipper status did not have independent associations with LVH (Table 2). The r^2^ of the model was 0.573, and Homer Lemeshow goodness-of-fit test indicated the model was a good fit (*p* = 0.335).

**Table 2.** Binary logistic regression analysis assessing factors independently associated with LVH.

MODEL 1	OR	95% confidence interval	*p* Value
**MBPS**	**6.625**	**1.048–41.882**	**0.044**
Age (years)	1.133	1.037–1.238	0.06
Average systolic BP (mmHg)	1.047	0.982–1.117	0.161
MODEL 2			
Dipper status	2.254	0.467–10.869	0.311
**Age (years)**	**1.160**	**1.065–1.263**	**0.001**
Average systolic BP (mmHg)	1.066	0.997–1.139	0.06

MBPS: morning blood pressure surge; RAS: renin-angiotensin-aldosterone system; BMI: body mass index; HDL: high-density lipoprotein; LDL: low-density lipoprotein; BP: blood pressure.

Adjusted r^2^ of MODEL 1 = 0.623, Homer Lemeshow goodness-of-fit *p* = 0.174; adjusted r^2^ of MODEL 2 = 0.573, Homer Lemeshow goodness-of-fit *p* = 0.335; The bold values represents as statistically significant *p* <.05 values.

## Discussion/conclusion

In the present study, we have shown, for the first time, a close relationship between prewaking MBPS and LVH in ADPKD patients with preserved renal function. There was also an independent association between higher age and LVH. Patients with MBPS had a higher mean LVMI value and also a higher rate of LVH. The presence of MBPS was also associated with worse endothelial functions, as shown by a higher RWT, a lower FMD, and a tendency toward higher CIMT.

In numerous studies, these CV abnormalities have been reported in ADPKD patients, but no clear etiology has been found until now. Although the main characteristics of ADPKD are cyst development throughout the body, noncystic findings such as increased sympathetic activation, early hypertension, and ED are not rare, and the cause of these abnormalities is not clear.

We hypothesize that increased sympathetic activity, especially in the early hours of awakening, may partly explain both prominent LVH and ED, which are prevalent in these patients [2,15]. CV alteration with declining renal function is a well-known abnormality in patients with CKD of different etiology [16]. CV abnormalities in ADPKD patients are slightly different from other causes of CKD. First, morphology and related defects such as cyst compression to the vascular structure are unique to ADPKD, and CV abnormalities such as ED and LVH may begin before kidney function declines. Our patients were seemingly have controlled blood pressure measurements in their out-patient follow-up, but 24-h monitoring demonstrated HT.

This finding was consistent with previous studies [17]. Furthermore, HT and LVH are very common in patients with ADPKD. In hypertensive populations, LVH is correlated with the degree of HT and aging, but interestingly, in ADPKD patients, LVH has been demonstrated in both young and normotensive patients [18]. Some studies reported the relation of LVH in ADPKD with cyst volume and HT [19]. However, increased cyst volume and accompanying pressure are not sufficient to explain HT and LVH when we consider young patients. In children with ADPKD, HT, and LVH are reported before significant cyst development [2]. In ADPKD patients, HT may partly explain the increased prevalence of LVH, but in the absence of HT, the presence of LVH is still a mystery. Our results show that during 24-h monitoring, the hypertensive or well controlled HT ADPKD patients with remarkable morning surge have more CV damage such as LVH, and ED as an early sign of atherosclerosis. In the hypertensive population, a non-dipper form of HT has been linked with increased adrenergic activity and decreased vagal activity during sleep, and found correlated with LVH [20,21]. In ADPKD patients, increased morning BP is a consequence of sympathetic nervous system activity (SSA) and may be partly responsible for LVH and early vascular abnormalities. In the early morning, blood pressure rises in response to the natural activation of the sympathetic nervous system in the circadian rhythm; but this response may be exaggerated in patients with increased sympathetic activity [21]. Valero et al. [22] reported that the nocturnal decrease in blood pressure was blunted in young patients with ADPKD, and 24-h systolic BP was the primary determinant of LVH; however, this trial did not investigate MBPS. Another point that should be emphasized in these patients is that pain should be assessed since nighttime pain may cause increased SSA during the night and may lead to adverse CV outcomes by increasing sympathetic activity. There are seasonal variations in the incidence of severe cardiac events with increased incidence in the winter. This is consistent with finding that sympathetic nerve activity varies along the seasons, with peak levels evident in the winter [23]. However, we did not carry out our study in a particular season.

Chronic pain is usually the result of increased cyst distension, which may activate the renal efferent sympathetic nerves. Hypertensive ADPKD patients with normal renal function have increased sympathetic activity, which partly explains HT in the early period of the disease [24]. Here, we found that LVH and FMD were prevalent among ADPKD patients with preserved renal functions. FMD is a result of nitric oxide (NO) mediation of vascular dilatation and decreased FMD is thought to decrease the availability of NO. In a study, although the number of patients was limited, ED was shown in early ADPKD patients before the development of HT. Increased activation of the RAS and decreased expression of PC1 or PC2 on vascular endothelium have been put forward as reasons for early ED in these patients [25,26]. However, the relation between MBPS and ED has not been investigated until now. In other populations, MBPS has been determined as a significant determinant of LVH; however, the relation with ED was not investigated at the same time. In our study, both LVH and ED (as shown by decreased FMD value) were significantly associated with the presence of MBPS in early ADPKD patients.

MBPS is multifactorial and also may be affected by environmental factors. Although high salt intake is a risk factor for HT no relation has been found between the MBPS and the rate of sodium consumption until now [27].

Our study has certain limitations, including the small sample size and cross-sectional and observational design. Another limitation of the present study is the fact that we did not evaluate the effects of seasonal variations, sodium consumption or excretion rate, alcohol consumption, stress or pain level, or sleep apnea, all of which may influence the presence and extent of MBPS. In the regression analysis, we did not include all factors that could potentially affect left ventricular hypertrophy, such as hemoglobin and albumin. Lack of more sophisticated and accurate measurement methods for left ventricular mass, such as magnetic resonance imaging, is another limitation of this study.

## Conclusions

To our knowledge, this study is the first one to investigate the effects of MBPS in ADPKD patients with preserved renal functions. The findings of this study suggest an independent association between LVH and MBPS and also between LVH and age in ADPKD patients. MBPS also seemed to be associated with worse endothelial functions, as shown by higher RWT, lower FMD, and a tendency toward higher CIMT. However, our results are not generalizable to all ADPKD patients because of the cross-sectional nature and limited sample size. It may be worth investigating the effects of reducing MBPS on the development of LVH and ED in patients with early ADPKD patients in randomized controlled studies.

## References

[1] Chebib FT, Torres VE. Autosomal dominant polycystic kidney disease: core curriculum 2016. Am J Kidney Dis. 2016;67:792–810. 26530876 10.1053/j.ajkd.2015.07.037 PMC4837006

[2] Ecder T. Cardiovascular complications in autosomal dominant polycystic kidney disease. Curr Hypertens Rev. 2013;9:2–11. 23971638 10.2174/1573402111309010002

[3] Cadnapaphornchai MA, McFann K, Strain JD, et al. Increased left ventricular mass in children with autosomal dominant polycystic kidney disease and borderline hypertension. Kidney Int. 2008;74:1192–1196. 18716604 10.1038/ki.2008.397 PMC2574635

[4] Li Kam Wa TC, Macnicol AM, Watson ML. Ambulatory blood pressure in hypertensive patients with autosomal dominant polycystic kidney disease. Nephrol Dial Transplant. 1997;12:2075–2080. 9351068 10.1093/ndt/12.10.2075

[5] Pucci G, Battista F, Anastasio F, et al. Morning pressor surge, blood pressure variability, and arterial stiffness in essential hypertension. J Hypertens. 2017;35:272–278. 28005700 10.1097/HJH.0000000000001153

[6] Okada Y, Galbreath MM, Shibata S, et al. Morning blood pressure surge is associated with arterial stiffness and sympathetic baroreflex sensitivity in hypertensive seniors. Am J Physiol Heart Circ Physiol. 2013;305:H793–H802. 23832695 10.1152/ajpheart.00254.2013 PMC3761347

[7] Turak O, Afsar B, Siriopol D, et al. Morning blood pressure surge as a predictor of development of chronic kidney disease. J Clin Hypertens (Greenwich). 2016;18:444–448. 26530334 10.1111/jch.12707 PMC8031569

[8] Pei Y, Obaji J, Dupuis A, et al. Unified criteria for ultrasonographic diagnosis of ADPKD. J Am Soc Nephrol. 2009;20:205–212. 18945943 10.1681/ASN.2008050507 PMC2615723

[9] Inker LA, Levey AS. Pro: Estimating GFR using the chronic kidney disease epidemiology collaboration (CKD-EPI) 2009 creatinine equation: the time for change is now. Nephrol Dial Transplant. 2013;28:1390–1396. 23780676 10.1093/ndt/gft003

[10] Kario K, Pickering TG, Umeda Y, et al. Morning surge in blood pressure as a predictor of silent and clinical cerebrovascular disease in elderly hypertensives: a prospective study. Circulation. 2003;107:1401–1406. 12642361 10.1161/01.cir.0000056521.67546.aa

[11] Bilo G, Grillo A, Guida V, et al. Morning blood pressure surge: pathophysiology, clinical relevance and therapeutic aspects. Integr Blood Press Control. 2018;11:47–56. 29872338 10.2147/IBPC.S130277 PMC5973439

[12] Lang RM, Bierig M, Devereux RB, et al., European Association of Echocardiography, European Society of Cardiology. Recommendations for chamber quantification. Eur J Echocardiogr. 2006;7:79–108. 16458610 10.1016/j.euje.2005.12.014

[13] Luciano RL, Dahl NK. Extra-renal manifestations of autosomal dominant polycystic kidney disease (ADPKD): considerations for routine screening and management. Nephrol Dial Transplant. 2014;29:247–254. 24215018 10.1093/ndt/gft437

[14] Wanner C, Amann K, Shoji T. The heart and vascular system in dialysis. Lancet. 2016;388:276–284. 27226133 10.1016/S0140-6736(16)30508-6

[15] de Almeida EA, de Oliveira EI, Lopes JA, et al. Ambulatory blood pressure measurement in young normotensive patients with autosomal dominant polycystic kidney disease. Rev Port Cardiol. 2007;26:235–243. 17549981

[16] Almeida EA, Oliveira EI, Lopes JA, et al. Tissue Doppler imaging in the evaluation of left ventricular function in young adults with autosomal dominant polycystic kidney disease. Am J Kidney Dis. 2006;47:587–592. 16564936 10.1053/j.ajkd.2005.12.023

[17] Chapman AB, Guay-Woodford LM, Grantham JJ, et al. Renal structure in early autosomal-dominant polycystic kidney disease (ADPKD): The Consortium for Radiologic Imaging Studies of Polycystic Kidney Disease (CRISP) cohort. Kidney Int. 2003;64:1035–1045. 12911554 10.1046/j.1523-1755.2003.00185.x

[18] Cuspidi C, Macca G, Sampieri L, et al. Target organ damage and non-dipping pattern defined by two sessions of ambulatory blood pressure monitoring in recently diagnosed essential hypertensive patients. J Hypertens. 2001;19:1539–1545. 11564972 10.1097/00004872-200109000-00004

[19] Peixoto AJ, White WB. Circadian blood pressure: clinical implications based on the pathophysiology of its variability. Kidney Int. 2007;71:855–860. 17377513 10.1038/sj.ki.5002130

[20] Valero FA, Martinez-Vea A, Bardaji A, et al. Ambulatory blood pressure and left ventricular mass in normotensive patients with autosomal dominant polycystic kidney disease. J Am Soc Nephrol. 1999;10:1020–1026. 10232688 10.1681/ASN.V1051020

[21] Cui J, Muller MD, Blaha C, et al. Seasonal variation in muscle sympathetic nerve activity. Physiol Rep. 2015;3:e12492. 26265752 10.14814/phy2.12492 PMC4562578

[22] Klein IH, Ligtenberg G, Oey PL, et al. Sympathetic activity is increased in polycystic kidney disease and is associated with hypertension. J Am Soc Nephrol. 2001;12:2427–2433. 11675419 10.1681/ASN.V12112427

[23] Kallakuri S, Yu JA, Li J, et al. Endothelial cilia are essential for developmental vascular integrity in zebrafish. J Am Soc Nephrol. 2015;26:864–875. 25214579 10.1681/ASN.2013121314 PMC4378100

[24] Lai S, Petramala L, Mastroluca D, et al. Hyperaldosteronism and cardiovascular risk in patients with autosomal dominant polycystic kidney disease. Medicine (Baltimore). 2016;95:e4175. 27442639 10.1097/MD.0000000000004175 PMC5265756

[25] van der Stouwe JG, Carmeli C, Aeschbacher S, et al. Association of 24-hour blood pressure with urinary sodium excretion in healthy adults. Am J Hypertens. 2018;31:784–791. 29481641 10.1093/ajh/hpy031

[26] Lee HT, Park JK, Choi SY, et al. Mediating effects of nocturnal blood pressure and morning surge on the contributions of arterial stiffness and sodium intake to morning blood pressure: a path analysis. Blood Press. 2016;25:28–35. 26415624 10.3109/08037051.2016.1091157

[27] Omboni S. Does dietary salt loading impair ambulatory blood pressure variability? As yet an unresolved issue. Am J Hypertens. 2020;33:405–406. 32080715 10.1093/ajh/hpaa028

